# Supplementary material for: Membrane-mediated dimerization potentiates PIP5K lipid kinase activity
Source: eLife. 2022 Aug 17;11:e73747. doi: 10.7554/eLife.73747 (PMC9470164; doi:10.7554/eLife.73747)
Supplement: Supplementary file 1. — Description of plasmids used for recombinant protein expression, purification, and transient transfection. [file elife-73747-supp1.docx]

**SUPPLEMENTAL FILE 1**

This document contains a list of plasmids used for recombinant protein expression and purification, as well as plasmids transfected into HEK293T cells for the single molecule cell lysate experiments. Following the list of plasmids are the primary amino acid sequences of each protein used in the study. Redundancies and mutant variants are not shown. Because several recombinantly expressed proteins are truncations of the full-length protein, this document also contains the full-length protein sequences retrieved from Uniprot (https://www.uniprot.org/).

Plasmids used for recombinant protein expression, purification, and transient transfection


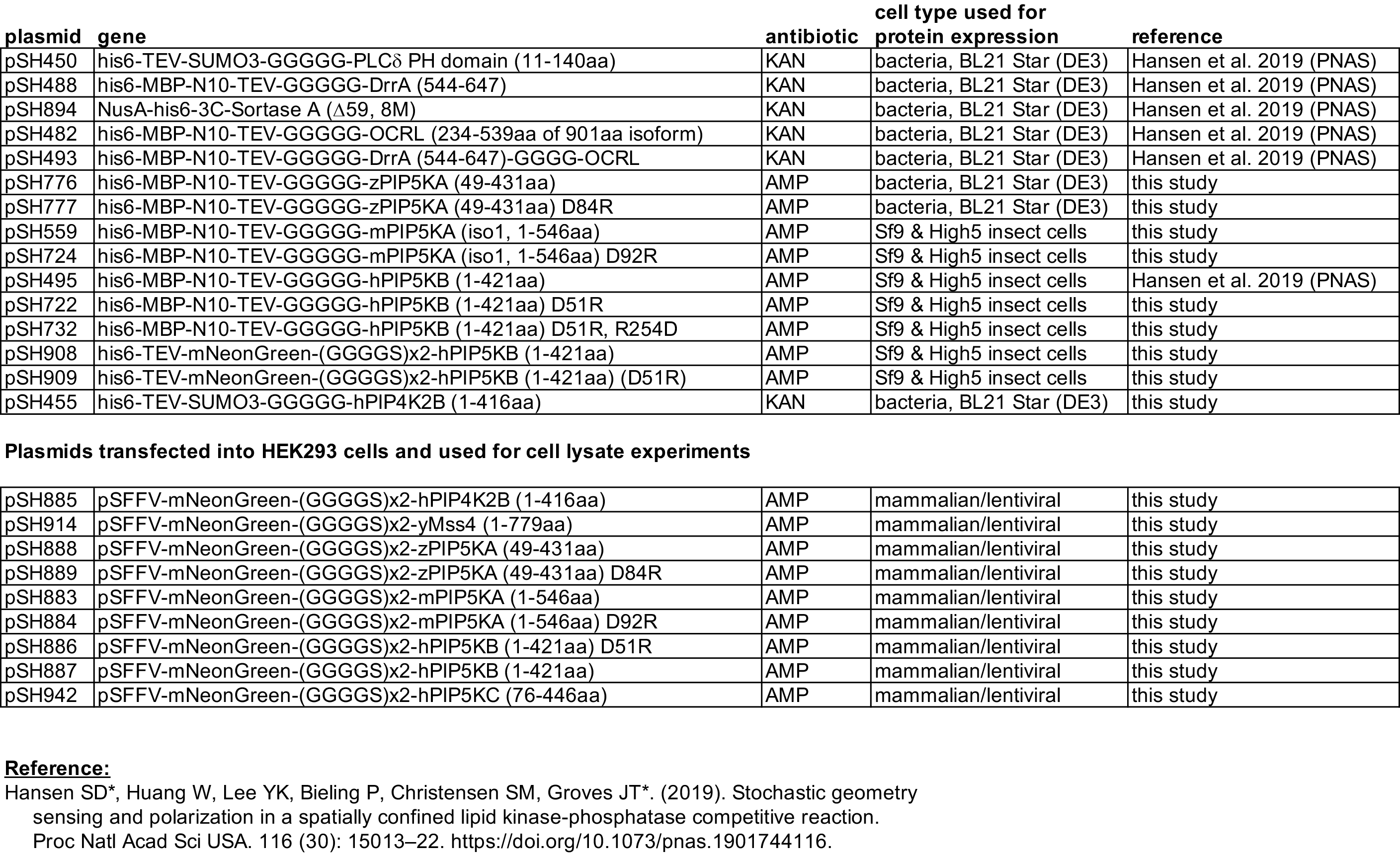


The following tags were cleaved off the recombinant proteins and removed during the purification:

**his6-TEV-SUMO3 (**↓= site of SUMO protease cleavage)

MKHHHHHHPMSDYDIPTTENLYFQGAMGNDHINLKVAGQDGSVVQFKIKRHTPLSKLMKAYCERQGLSMRQIRFRFDGQPINETDTPAQLEMEDEDTIDVFQQQTGG↓GGGG

**his6-MBP-N10-TEV (**↓= site of TEV protease cleavage)

MGSSHHHHHHGSSMKIEEGKLVIWINGDKGYNGLAEVGKKFEKDTGIKVTVEHPDKLEEKFPQVAATGDGPDIIFWAHDRFGGYAQSGLLAEITPDKAFQDKLYPFTWDAVRYNGKLIAYPIAVEALSLIYNKDLLPNPPKTWEEIPALDKELKAKGKSALMFNLQEPYFTWPLIAADGGYAFKYENGKYDIKDVGVDNAGAKAGLTFLVDLIKNKHMNADTDYSIAEAAFNKGETAMTINGPWAWSNIDTSKVNYGVTVLPTFKGQPSKPFVGVLSAGINAASPNKELAKEFLENYLLTDEGLEAVNKDKPLGAVALKSYEEELAKDPRIAATMENAQKGEIMPNIPQMSAFWYAVRTAVINAASGRQTVDEALKDAQTNSSSNNNNNNNNNNLGIEENLYFQ↓GGGGG

Shown below are the plasmid #’s and names of the full-length recombinant proteins that were expressed and purified in this study. The affinity and solubility tags that are underlined in the gene names were cleaved off the indicated protein and removed through purification. The primary amino acid sequence shown below represents the final purified product.

**pSH450, his6-TEV-SUMO3-GGGG-PLCδ PH domain**

…GGGGTSHGLQDDEDLQALLKGSQLLKVKSSSWRRERFYKLQEDCKTIWQESRKVMRTPESQLFSIEDIQEVRMGHRTEGLEKFARDVPEDRCFSIVFKDQRNTLDLIAPSPADAQHWVLGLHKIIHHSGSMDQRQKGS*

**pSH488, his6-MBP-N10-TEV-GGGGG-DrrA (544-647)**

…GGGGGTSRYTASTENFKNVKEKYQQMRGDALKTEILADFKDKLAEATDEQSLKQIVAELKSKDEYRILAKGQGLTTQLLGLKTSSVSSFEKMVEETRESIKSQERQTIKIK*

**pSH894, NusA-his6-3C-Sortase A (Δ59, 8M)**

…GPMGMQAKPQIPKDKSKVAGYIEIPDADIKEPVYPGPATREQLNRGVSFAEENESLDDQNISIAGHTFIGRPNYQFTNLKAAKKGSMVYFKVGNETRKYKMTSIRNVKPTAVGVLDEQKGKDKQLTLITCDDLNRETGVWETRKIFVATEVK*

**pSH482, his6-MBP-N10-TEV-GGGGG-OCRL**

…GGGGGTSYVNIQTFRFFVGTWNVNGQSPDSGLEPWLNCDPNPPDIYCIGFQELDLSTEAFFYFESVKEQEWSMAVERGLHSKAKYKKVQLVRLVGMMLLIFARKDQCRYIRDIATETVGTGIMGKMGNKGGVAVRFVFHNTTFCIVNSHLAAHVEDFERRNQDYKDICARMSFVVPNQTLPQLNIMKHEVVIWLGDLNYRLCMPDANEVKSLINKKDLQRLLKFDQLNIQRTQKKAFVDFNEGEIKFIPTYKYDSKTDRWDSSGKCRVPAWCDRILWRGTNVNQLNYRSHMELKTSDHKPVSALFHIGVKVVD*

**pSH493, his6-MBP-N10-TEV-GGGGG-DrrA (544-647)-GGGGG-OCRL**

…GGGGGTSRYTASTENFKNVKEKYQQMRGDALKTEILADFKDKLAEATDEQSLKQIVAELKSKDEYRILAKGQGLTTQLLGLKTSSVSSFEKMVEETRESIKSQERQTIKIKGSGGGGGTSYVNIQTFRFFVGTWNVNGQSPDSGLEPWLNCDPNPPDIYCIGFQELDLSTEAFFYFESVKEQEWSMAVERGLHSKAKYKKVQLVRLVGMMLLIFARKDQCRYIRDIATETVGTGIMGKMGNKGGVAVRFVFHNTTFCIVNSHLAAHVEDFERRNQDYKDICARMSFVVPNQTLPQLNIMKHEVVIWLGDLNYRLCMPDANEVKSLINKKDLQRLLKFDQLNIQRTQKKAFVDFNEGEIKFIPTYKYDSKTDRWDSSGKCRVPAWCDRILWRGTNVNQLNYRSHMELKTSDHKPVSALFHIGVKVVD*

**pSH455, his6-TEV-SUMO3-GGGGG-hPIP4K2B (1-416aa)**

…GGGGGTSMSSNCTSTTAVAVAPLSASKTKTKKKHFVCQKVKLFRASEPILSVLMWGVNHTINELSNVPVPVMLMPDDFKAYSKIKVDNHLFNKENLPSRFKFKEYCPMVFRNLRERFGIDDQDYQNSVTRSAPINSDSQGRCGTRFLTTYDRRFVIKTVSSEDVAEMHNILKKYHQFIVECHGNTLLPQFLGMYRLTVDGVETYMVVTRNVFSHRLTVHRKYDLKGSTVAREASDKEKAKDLPTFKDNDFLNEGQKLHVGEESKKNFLEKLKRDVEFLAQLKIMDYSLLVGIHDVDRAEQEEMEVEERAEDEECENDGVGGNLLCSYGTPPDSPGNLLSFPRFFGPGEFDPSVDVYAMKSHESSPKKEVYFMAIIDILTPYDTKKKAAHAAKTVKHGAGAEISTVNPEQYSKRFNEFMSNILT*

Residues highlighted in **blue** represent the conserved aspartic acid that helps mediate dimerization of PIP5K.

**pSH776, his6-MBP-N10-TEV-GGGGG-zPIP5KA (49-431aa)**

…GGGGGTGETTYKKTTSSALKGAIQLGITHSVGSLSQKPERDVLMQDFEVVESIFFPSQGSSSTPGHHHGDFKFKTYAPIAFRYFREMFGIRPDDYLYSLCNEPLIELSNPGASGSLFYVSSDDEFIIKTVQHKEAEFLQTLLPGYFMNLNQNMRTLLPKFYGLYCVQADGKNIRIVVMNNLLPRAVPMHLKFDLKGSTYKRRASPKERSKGVPTYKDLDFMQDMPEGILLENDHYTALSRTMQRDCRVLQSFKIMDYSLLVGIHILHRAGEEASTAVPDTQKKGQGQKPLYCTAIESIQGESKSKTSPQPYESMGGIPAFNSKGERLLVFIGIIDILQSYRLVKKLEHSWKALLHDGDTVSVHRPSFYADRFQKFMCSTVFRKSQLKT*

**pSH559, his6-MBP-N10-TEV-GGGGG-mPIP5KA (iso1, 1-546aa)**

…GGGGGTSMASASSGPAAAGFSSLDAGAPAGTAAASGIKRATVSEGPSASVMPVKKIGHRSVDSSGETTYKKTTSSALKGAIQLGITHTVGSLSTKPERDVLMQDFYVVESIFFPSEGSNLTPAHHYNDFRFKTYAPVAFRYFRELFGIRPDDYLYSLCSEPLIELSNSGASGSLFYVSSDDEFIIKTVQHKEAEFLQKLLPGYYMNLNQNPRTLLPKFYGLYCVQAGGKNIRIVVMNNLLPRSVKMHMKYDLKGSTYKRRASQKEREKTLPTFKDLDFLQDIPDGLFLDADMYSALCKTLQRDCLVLQSFKIMDYSLLMSIHNMDHAQREPTSNDTQYSADTRRPAPQKALYSTAMESIQGEARRGGTVETEDHMGGIPARNNKGERLLLYIGIIDILQSYRFVKKLEHSWKALVHDGDTVSVHRPGFYAERFQRFMCNTVFKKIPLKPSPTKKFRSGPSFSRRSGPSGNSCTSQLMASGEHRAQVTTKAEVEPDVHLGRPDVLPQTPPLEEISEGSPVPGPSFSPVVGQPLQILNLSSTLEKLDVAESEFTH*

**pSH495, his6-MBP-N10-TEV-GGGGG-hPIP5KB (1-421aa)**

…GGGGGTSMSSAAENGEAAPGKQNEEKTYKKTASSAIKGAIQLGIGYTVGNLTSKPERDVLMQDFYVVESVFLPSEGSNLTPAHHYPDFRFKTYAPLAFRYFRELFGIKPDDYLYSICSEPLIELSNPGASGSLFFVTSDDEFIIKTVQHKEAEFLQKLLPGYYMNLNQNPRTLLPKFYGLYCMQSGGINIRIVVMNNVLPRSMRMHFTYDLKGSTYKRRASRKEREKSNPTFKDLDFLQDMHEGLYFDTETYNALMKTLQRDCRVLESFKIMDYSLLLGIHFLDHSLKEKEEETPQNVPDAKRTGMQKVLYSTAMESIQGPGKSGDGIITENPDTMGGIPAKSHRGEKLLLFMGIIDILQSYRLMKKLEHSWKALVYDGDTVSVHRPSFYADRFLKFMNSRVFKKIQALKASPSKKRCNSIAALKATS*

**pSH908, his6-TEV-mNeonGreen-(GGGGS)x2-hPIP5KB (1-421aa)**

**…**SNTGMVSKGEEDNMASLPATHELHIFGSINGVDFDMVGQGTGNPNDGYEELNLKSTKGDLQFSPWILVPHIGYGFHQYLPYPDGMSPFQAAMVDGSGYQVHRTMQFEDGASLTVNYRYTYEGSHIKGEAQVKGTGFPADGPVMTNSLTAADWCRSKKTYPNDKTIISTFKWSYTTGNGKRYRSTARTTYTFAKPMAANYLKNQPMYVFRKTELKHSKTELNFKEWQKAFTDVMGMDELYKGGGGSGGGGSTSMSSAAENGEAAPGKQNEEKTYKKTASSAIKGAIQLGIGYTVGNLTSKPERDVLMQDFYVVESVFLPSEGSNLTPAHHYPDFRFKTYAPLAFRYFRELFGIKPDDYLYSICSEPLIELSNPGASGSLFFVTSDDEFIIKTVQHKEAEFLQKLLPGYYMNLNQNPRTLLPKFYGLYCMQSGGINIRIVVMNNVLPRSMRMHFTYDLKGSTYKRRASRKEREKSNPTFKDLDFLQDMHEGLYFDTETYNALMKTLQRDCRVLESFKIMDYSLLLGIHFLDHSLKEKEEETPQNVPDAKRTGMQKVLYSTAMESIQGPGKSGDGIITENPDTMGGIPAKSHRGEKLLLFMGIIDILQSYRLMKKLEHSWKALVYDGDTVSVHRPSFYADRFLKFMNSRVFKKIQALKASPSKKRCNSIAALKATS*

**pSH914, mNeonGreen-(GGGGS)x2-yMss4 (1-779aa)**

MVSKGEEDNMASLPATHELHIFGSINGVDFDMVGQGTGNPNDGYEELNLKSTKGDLQFSPWILVPHIGYGFHQYLPYPDGMSPFQAAMVDGSGYQVHRTMQFEDGASLTVNYRYTYEGSHIKGEAQVKGTGFPADGPVMTNSLTAADWCRSKKTYPNDKTIISTFKWSYTTGNGKRYRSTARTTYTFAKPMAANYLKNQPMYVFRKTELKHSKTELNFKEWQKAFTDVMGMDELYKGGGGSGGGGSTSMSVLRSQPPSVVPLHLTTSTSRKTEQEPSLLHSAIIERHQDRSVPNSNSNPDSNHRIKKDRNNHTSYHSSSNSESNMESPRLSDGESSTPTSIEELNPTINNSRLVKRNYSISIDPLHDNSNNNTDDDHPNTITSPRPNSTSNKEMQKYSFPEGKESKKITTPSLNSNNCLDLDNSSLVHTDSYIQDLNDDHILLNKRVSRRSSRISAVTATSTTIKQRRNTQDSNLPNIPFHASKHSQILPMDDSDVIKLANGDTSMKPNSATKISHSMTSLPLHPLPQPSQKSKQYHMISKSTTSLPPENDHYYQHSRGTNHNHAANAAAVNNNTTTTTAATGLKRSESATAEIKKMRQSLLHKREMKRKRKTFLVDDDRVLIGNKVSEGHVNFIIAYNMLTGIRVAVSRCSGIMKPLTPADFRFTKKLAFDYHGNELTPSSQYAFKFKDYCPEVFRELRALFGLDPADYLVSLTSKYILSELNSPGKSGSFFYYSRDYKYIIKTIHHSEHIHLRKHIQEYYNHVRDNPNTLICQFYGLHRVKMPISFQNKIKHRKIYFLVMNNLFPPHLDIHITYDLKGSTWGRFTNLDKERLAKDRSYRPVMKDLNWLEEGQKIKFGPLKKKTFLTQLKKDVELLAKLNTMDYSLLIGIHDINKAKEDDLQLADTASIEEQPQTQGPIRTGTGTVVRHFFREFEGGIRASDQFNNDVDLIYYVGIIDFLTNYSVMKKLETFWRSLRHDTKLVSAIPPRDYANRFYEFIEDSVDPLPQKKTQSSYRDDPNQKNYKD*

**pSH942, mNeonGreen-(GGGGS)x2-hPIP5KC (76-446aa)**

MVSKGEEDNMASLPATHELHIFGSINGVDFDMVGQGTGNPNDGYEELNLKSTKGDLQFSPWILVPHIGYGFHQYLPYPDGMSPFQAAMVDGSGYQVHRTMQFEDGASLTVNYRYTYEGSHIKGEAQVKGTGFPADGPVMTNSLTAADWCRSKKTYPNDKTIISTFKWSYTTGNGKRYRSTARTTYTFAKPMAANYLKNQPMYVFRKTELKHSKTELNFKEWQKAFTDVMGMDELYKGGGGSGGGGSTSLKGAIQLGIGYTVGHLSSKPERDVLMQDFYVVESIFFPSEGSNLTPAHHFQDFRFKTYAPVAFRYFRELFGIRPDDYLYSLCNEPLIELSNPGASGSLFYVTSDDEFIIKTVMHKEAEFLQKLLPGYYMNLNQNPRTLLPKFYGLYCVQSGGKNIRVVVMNNILPRVVKMHLKFDLKGSTYKRRASKKEKEKSFPTYKDLDFMQDMPEGLLLDADTFSALVKTLQRDCLVLESFKIMDYSLLLGVHNIDQHERERQAQGAQSTSDEKRPVGQKALYSTAMESIQGGAARGEAIESDDTMGGIPAVNGRGERLLLHIGIIDILQSYRFIKKLEHTWKALVHDGDTVSVHRPSFYAERFFKFMSNTVFRK*

Full-length protein sequences retrieved from Uniprot. Note that truncated variants of these full-length proteins were recombinantly expressed and purified in this study.

**hPIP4KB (full-length kinase = 1-416aa)**

**Uniprot #P78356 (https://www.uniprot.org/uniprotkb/P78356/entry)**

**human phosphatidylinositol 5-phosphate 4-kinase, type 2, beta isoform 1**

MSSNCTSTTAVAVAPLSASKTKTKKKHFVCQKVKLFRASEPILSVLMWGVNHTINELSNVPVPVMLMPDDFKAYSKIKVDNHLFNKENLPSRFKFKEYCPMVFRNLRERFGIDDQDYQNSVTRSAPINSDSQGRCGTRFLTTYDRRFVIKTVSSEDVAEMHNILKKYHQFIVECHGNTLLPQFLGMYRLTVDGVETYMVVTRNVFSHRLTVHRKYDLKGSTVAREASDKEKAKDLPTFKDNDFLNEGQKLHVGEESKKNFLEKLKRDVEFLAQLKIMDYSLLVGIHDVDRAEQEEMEVEERAEDEECENDGVGGNLLCSYGTPPDSPGNLLSFPRFFGPGEFDPSVDVYAMKSHESSPKKEVYFMAIIDILTPYDTKKKAAHAAKTVKHGAGAEISTVNPEQYSKRFNEFMSNILT*

**zPIP5KA (full-length kinase = 1-592aa)**

**Uniprot # A0A2R8QMJ9** (**https://www.uniprot.org/uniprotkb/A0A2R8QMJ9/entry)**

**zebrafish phosphatidylinositol 4-phosphate 5-kinase, type 1, alpha**

MASACPPDTGLSAPPGSSGVRKMTITEGPGSSQSMKKTIGHRGVDPTGETTYKKTTSSALKGAIQLGIAHTVGSLSQKAERDVLMQDFYVVESIFFPSEGSNLTPAHHHGDFRFKTYAPIAFRYFRELFGIRPDDYLYSLCNDPLIELSNPGASGSIFYVTSDDEFIIKTVMHKEAEFLQKLLPGYFMNLNQNKRTLLPKFYGLYCVQAGGKNIRIVVMNNLLPRSVPMHLKYDLKGSTYKRRASPKERDKSVPTYKDLDFIQDMPEGIQLEPDNYNALCKTIQRDCLLLQSFKIMDYSLLVGVHNTDLASRERAGVVEGGGSEGTVTPDHRRPQIQKALYSTAMESIQGEAKGKGTLETEDQWGGIPARNSRGERILVYIGIIDILQSYRFIKKLEHSWKALVHDGDTVSVHRPGFYAERFQRFMCNTVFKKTLKSSPSKKSRSGCSSVVRRLPMGSTASAPGSQTLADARLVYRTHLNQPDLEGESGMPSDRPDLLPQTDPLAGSSSEFAATNVSCSSPGSTRMTSSSPPQRSVGVEVHKSAVTEPDNSTLHSTGAECLDEQLSNEDAISLKDIIPETDICFVCTNLHIC*

**mPIP5KA (full-length kinase = 1-546aa)**

**Uniprot #P70182 (https://www.uniprot.org/uniprotkb/P70182/entry)**

**mouse phosphatidylinositol 4-phosphate 5-kinase, type 1, alpha isoform 1**

MASASSGPAAAGFSSLDAGAPAGTAAASGIKRATVSEGPSASVMPVKKIGHRSVDSSGETTYKKTTSSALKGAIQLGITHTVGSLSTKPERDVLMQDFYVVESIFFPSEGSNLTPAHHYNDFRFKTYAPVAFRYFRELFGIRPDDYLYSLCSEPLIELSNSGASGSLFYVSSDDEFIIKTVQHKEAEFLQKLLPGYYMNLNQNPRTLLPKFYGLYCVQAGGKNIRIVVMNNLLPRSVKMHMKYDLKGSTYKRRASQKEREKTLPTFKDLDFLQDIPDGLFLDADMYSALCKTLQRDCLVLQSFKIMDYSLLMSIHNMDHAQREPTSNDTQYSADTRRPAPQKALYSTAMESIQGEARRGGTVETEDHMGGIPARNNKGERLLLYIGIIDILQSYRFVKKLEHSWKALVHDGDTVSVHRPGFYAERFQRFMCNTVFKKIPLKPSPTKKFRSGPSFSRRSGPSGNSCTSQLMASGEHRAQVTTKAEVEPDVHLGRPDVLPQTPPLEEISEGSPVPGPSFSPVVGQPLQILNLSSTLEKLDVAESEFTH*

**hPIP5KB (full-length kinase = 1-540aa)**

**Uniprot #O14986 (https://www.uniprot.org/uniprotkb/O14986/entry)**

**human phosphatidylinositol 4-phosphate 5-kinase, type 1, beta isoform 2**

MSSAAENGEAAPGKQNEEKTYKKTASSAIKGAIQLGIGYTVGNLTSKPERDVLMQDFYVVESVFLPSEGSNLTPAHHYPDFRFKTYAPLAFRYFRELFGIKPDDYLYSICSEPLIELSNPGASGSLFFVTSDDEFIIKTVQHKEAEFLQKLLPGYYMNLNQNPRTLLPKFYGLYCMQSGGINIRIVVMNNVLPRSMRMHFTYDLKGSTYKRRASRKEREKSNPTFKDLDFLQDMHEGLYFDTETYNALMKTLQRDCRVLESFKIMDYSLLLGIHFLDHSLKEKEEETPQNVPDAKRTGMQKVLYSTAMESIQGPGKSGDGIITENPDTMGGIPAKSHRGEKLLLFMGIIDILQSYRLMKKLEHSWKALVYDGDTVSVHRPSFYADRFLKFMNSRVFKKIQALKASPSKKRCNSIAALKATSQEIVSSISQEWKDEKRDLLTEGQSFSSLDEEALGSRHRPDLVPSTPSLFEAASLATTISSSSLYVNEHYPHDRPTLYSNSKGLPSSSTFTLEEGTIYLTAEPNTLEVQDDNASVLDVYL*

**hPIP5KC (full-length kinase = 1-668aa)**

**Uniprot #O60331 (https://www.uniprot.org/uniprotkb/O60331/entry)**

**human phosphatidylinositol 4-phosphate 5-kinase, type 1, gamma isoform 1**

MELEVPDEAESAEAGAVPSEAAWAAESGAAAGLAQKKAAPTEVLSMTAQPGPGHGKKLGHRGVDASGETTYKKTTSSTLKGAIQLGIGYTVGHLSSKPERDVLMQDFYVVESIFFPSEGSNLTPAHHFQDFRFKTYAPVAFRYFRELFGIRPDDYLYSLCNEPLIELSNPGASGSLFYVTSDDEFIIKTVMHKEAEFLQKLLPGYYMNLNQNPRTLLPKFYGLYCVQSGGKNIRVVVMNNILPRVVKMHLKFDLKGSTYKRRASKKEKEKSFPTYKDLDFMQDMPEGLLLDADTFSALVKTLQRDCLVLESFKIMDYSLLLGVHNIDQHERERQAQGAQSTSDEKRPVGQKALYSTAMESIQGGAARGEAIESDDTMGGIPAVNGRGERLLLHIGIIDILQSYRFIKKLEHTWKALVHDGDTVSVHRPSFYAERFFKFMSNTVFRKNSSLKSSPSKKGRGGALLAVKPLGPTAAFSASQIPSEREEAQYDLRGARSYPTLEDEGRPDLLPCTPPSFEEATTASIATTLSSTSLSIPERSPSETSEQPRYRRRTQSSGQDGRPQEEPPAEEDLQQITVQVEPACSVEIVVPKEEDAGVEASPAGASAAVEVETASQASDEEGAPASQASDEEDAPATDIYFPTDERSWVYSPLHYSAQAPPASDGESDT*

**yMss4 (full-length kinase = 1-779aa)**

**Uniprot #P38994 (https://www.uniprot.org/uniprotkb/P38994/entry)**

**yeast multicopy suppressor of Stt4 or phosphatidylinositol 4-phosphate 5-kinase** MSVLRSQPPSVVPLHLTTSTSRKTEQEPSLLHSAIIERHQDRSVPNSNSNPDSNHRIKKDRNNHTSYHSSSNSESNMESPRLSDGESSTPTSIEELNPTINNSRLVKRNYSISIDPLHDNSNNNTDDDHPNTITSPRPNSTSNKEMQKYSFPEGKESKKITTPSLNSNNCLDLDNSSLVHTDSYIQDLNDDHILLNKRVSRRSSRISAVTATSTTIKQRRNTQDSNLPNIPFHASKHSQILPMDDSDVIKLANGDTSMKPNSATKISHSMTSLPLHPLPQPSQKSKQYHMISKSTTSLPPENDHYYQHSRGTNHNHAANAAAVNNNTTTTTAATGLKRSESATAEIKKMRQSLLHKREMKRKRKTFLVDDDRVLIGNKVSEGHVNFIIAYNMLTGIRVAVSRCSGIMKPLTPADFRFTKKLAFDYHGNELTPSSQYAFKFKDYCPEVFRELRALFGLDPADYLVSLTSKYILSELNSPGKSGSFFYYSRDYKYIIKTIHHSEHIHLRKHIQEYYNHVRDNPNTLICQFYGLHRVKMPISFQNKIKHRKIYFLVMNNLFPPHLDIHITYDLKGSTWGRFTNLDKERLAKDRSYRPVMKDLNWLEEGQKIKFGPLKKKTFLTQLKKDVELLAKLNTMDYSLLIGIHDINKAKEDDLQLADTASIEEQPQTQGPIRTGTGTVVRHFFREFEGGIRASDQFNNDVDLIYYVGIIDFLTNYSVMKKLETFWRSLRHDTKLVSAIPPRDYANRFYEFIEDSVDPLPQKKTQSSYRDDPNQKNYKD*

Additional sequences that were not used in this study, but are useful for comparing sequence homology between mouse and human PIP5KA/B.

**hPIP5KA (full-length kinase = 1-562aa)**

**Uniprot #Q99755 (https://www.uniprot.org/uniprotkb/Q99755/entry)**

**human phosphatidylinositol 4-phosphate 5-kinase, type 1, alpha**

MASASSGPSSSVGFSSFDPAVPSCTLSSAASGIKRPMASEVLEARQDSYISLVPYASGMPIKKIGHRSVDSSGETTYKKTTSSALKGAIQLGITHTVGSLSTKPERDVLMQDFYVVESIFFPSEGSNLTPAHHYNDFRFKTYAPVAFRYFRELFGIRPDDYLYSLCSEPLIELCSSGASGSLFYVSSDDEFIIKTVQHKEAEFLQKLLPGYYMNLNQNPRTLLPKFYGLYCVQAGGKNIRIVVMNNLLPRSVKMHIKYDLKGSTYKRRASQKEREKPLPTFKDLDFLQDIPDGLFLDADMYNALCKTLQRDCLVLQSFKIMDYSLLMSIHNIDHAQREPLSSETQYSVDTRRPAPQKALYSTAMESIQGEARRGGTMETDDHMGGIPARNSKGERLLLYIGIIDILQSYRFVKKLEHSWKALVHDGDTVSVHRPGFYAERFQRFMCNTVFKKIPLKPSPSKKFRSGSSFSRRAGSSGNSCITYQPSVSGEHKAQVTTKAEVEPGVHLGRPDVLPQTPPLEEISEGSPIPDPSFSPLVGETLQMLTTSTTLEKLEVAESEFTH*

**mPIP5KB (full-length kinase = 1-539aa)**

**Uniprot #P70181 (https://www.uniprot.org/uniprotkb/P70181/entry)**

**mouse phosphatidylinositol 4-phosphate 5-kinase, type 1, beta**

MSSTAENGDAVPGKQNEEKTYKKTASSAIKGAIQLGIGYTVGNLTSKPERDVLMQDFYVVESVFLPSEGSNLTPAHHYPDFRFKTYAPLAFRYFRELFGIKPDDYLYSICSEPLIELSNPGASGSLFFLTSDDEFIIKTVQHKEAEFLQKLLPGYYMNLNQNPRTLLPKFYGLYCMQSGGINIRIVVMNNVLPRAMRMHLTYDLKGSTYKRRASRKEREKPNPTFKDLDFLQDMHEGLYFDTETYNALMKTLQRDCRVLESFKIMDYSLLLGIHILDHSLKDKEEEPLQNVPDAKRPGMQKVLYSTAMESIQGPGKSADGIIAENPDTMGGIPAKSHKGEKLLLFMGIIDILQSYRLMKKLEHSWKALVYDGDTVSVHRPSFYADRFLKFMNSRVFKKIQALKASPSKKRCNSIAALKATSQEIVSSISQEWKDEKRDLLTEGQSFSSLDEEALGSRHRPDLVPSTPSLFEAASLATTISSSSLYVGEHYPHDRTTLYSNSKGLPSSSTFTLEEGTIYLTAEPNTLDLQDDASVLDVYL*
