# Supplementary material for: Membrane-mediated dimerization potentiates PIP5K lipid kinase activity
Source: eLife. 2022 Aug 17;11:e73747. doi: 10.7554/eLife.73747 (PMC9470164; doi:10.7554/eLife.73747)
Supplement: Transparent reporting form [file elife-73747-transrepform1.docx]

***eLife’s* transparent reporting form**

We encourage authors to provide detailed information *within their submission* to facilitate the interpretation and replication of experiments. Authors can upload supporting documentation to indicate the use of appropriate reporting guidelines for health-related research (see [EQUATOR Network](http://www.equator-network.org/%20)), life science research (see the [BioSharing Information Resource](https://biosharing.org/)), or the [ARRIVE guidelines](http://www.plosbiology.org/article/info:doi/10.1371/journal.pbio.1000412) for reporting work involving animal research. Where applicable, authors should refer to any relevant reporting standards documents in this form.

If you have any questions, please consult our Journal Policies and/or contact us: [editorial@elifesciences.org](mailto:editorial@elifesciences.org).

**Sample-size estimation**

- You should state whether an appropriate sample size was computed when the study was being designed
- You should state the statistical method of sample size computation and any required assumptions
- If no explicit power analysis was used, you should describe how you decided what sample (replicate) size (number) to use

Please outline where this information can be found within the submission (e.g., sections or figure legends), or explain why this information doesn’t apply to your submission:

Sample size in the context of single particle tracking analysis was determined based on the standards in the field. Specific details about sample size for single molecule dwell time data and step size distributions can be found in Figure legends, Table 1, and Table 2. General statements about sample size can be found in the Methods section titled, “Image analysis, curve fitting, and statistics.”

**Replicates**

- You should report how often each experiment was performed
- You should include a definition of biological versus technical replication
- The data obtained should be provided and sufficient information should be provided to indicate the number of independent biological and/or technical replicates
- If you encountered any outliers, you should describe how these were handled
- Criteria for exclusion/inclusion of data should be clearly stated
- High-throughput sequence data should be uploaded before submission, with a private link for reviewers provided (these are available from both GEO and ArrayExpress)

Please outline where this information can be found within the submission (e.g., sections or figure legends), or explain why this information doesn’t apply to your submission:

Biochemical assays were performed using mixture of technical and biological replicated. Specific details can be found in the Methods sections titled, “Image analysis, curve fitting, and statistics.” See the Methods section titled**,** “Single particle tracking,” for a detailed explanation of our analysis method. Information about replicate number can be found in the Figure legends, Table 1, and Table 2.

**Statistical reporting**

- Statistical analysis methods should be described and justified
- Raw data should be presented in figures whenever informative to do so (typically when N per group is less than 10)
- For each experiment, you should identify the statistical tests used, exact values of N, definitions of center, methods of multiple test correction, and dispersion and precision measures (e.g., mean, median, SD, SEM, confidence intervals; and, for the major substantive results, a measure of effect size (e.g., Pearson's r, Cohen's d)
- Report exact p-values wherever possible alongside the summary statistics and 95% confidence intervals. These should be reported for all key questions and not only when the p-value is less than 0.05.

Please outline where this information can be found within the submission (e.g., sections or figure legends), or explain why this information doesn’t apply to your submission:

No p-values were reported in this article. Where appropriate, error bars in the form of SD were included data plots. Statistics can be found in the Figure legends, Table 1, and Table 2.

(For large datasets, or papers with a very large number of statistical tests, you may upload a single table file with tests, Ns, etc., with reference to sections in the manuscript.)

**Group allocation**

- Indicate how samples were allocated into experimental groups (in the case of clinical studies, please specify allocation to treatment method); if randomization was used, please also state if restricted randomization was applied
- Indicate if masking was used during group allocation, data collection and/or data analysis

Please outline where this information can be found within the submission (e.g., sections or figure legends), or explain why this information doesn’t apply to your submission:

Not applicable for this study.

**Additional data files (“source data”)**

- We encourage you to upload relevant additional data files, such as numerical data that are represented as a graph in a figure, or as a summary table
- Where provided, these should be in the most useful format, and they can be uploaded as “Source data” files linked to a main figure or table
- Include model definition files including the full list of parameters used
- Include code used for data analysis (e.g., R, MatLab)
- Avoid stating that data files are “available upon request”

Please indicate the figures or tables for which source data files have been provided:

The following source data files are provided:

Figure 1 – Source Data 1; related to Figure 1C

Figure 1 – Source Data 2; related to Figure 1D

Figure 1 – Source Data 3; related to Figure 1E

Figure 1 – Source Data 4; related to Figure 1F

Figure 1 – Source Data 5; related to Figure 1G

Figure 1 – Source Data 6; related to Figure 1H

Figure 1 – Source Data 7; related to Figure 1I

Figure 1 – Source Data 8; related to Figure 1J

Figure 1 – Source Data 9; related to Figure 1–figure supplement 1

Figure 1 – Source Data 10; related to Figure 1–figure supplement 2

Figure 1 – Source Data 11; related to Figure 1–figure supplement 3

Figure 1 – Source Data 12; related to Figure 1–figure supplement 4

Figure 2 – Source Data 1; related to Figure 2B

Figure 2 – Source Data 2; related to Figure 2C

Figure 2 – Source Data 3; related to Figure 2F

Figure 2 – Source Data 4; related to Figure 2–figure supplement 1

Figure 2 – Source Data 5; related to Figure 2–figure supplement 2

Figure 2 – Source Data 6; related to Figure 2–figure supplement 3

Figure 3 – Source Data 1; related to Figure 3C

Figure 3 – Source Data 2; related to Figure 3D

Figure 3 – Source Data 3; related to Figure 3E

Figure 3 – Source Data 4; related to Figure 3F

Figure 3 – Source Data 5; related to Figure 3–figure supplement 1

Figure 4 – Source Data 1; related to Figure 4A

Figure 4 – Source Data 2; related to Figure 4C

Figure 4 – Source Data 3; related to Figure 4D

Figure 4 – Source Data 4; related to Figure 4F

Figure 4 – Source Data 5; related to Figure 4G

Figure 4 – Source Data 6; related to Figure 4H

Figure 4 – Source Data 7; related to Figure 4I

Figure 4 – Source Data 8; related to Figure 4–figure supplement 1

Figure 5 – Source Data 1; related to Figure 5A

Figure 5 – Source Data 2; related to Figure 5B

Figure 5 – Source Data 3; related to Figure 5C

Figure 5 – Source Data 4; related to Figure 5D

Figure 5 – Source Data 5; related to Figure 5F

Figure 5 – Source Data 6; related to Figure 5G

Figure 5 – Source Data 7; related to Figure 5H

Figure 5 – Source Data 8; related to Figure 5–figure supplement 1

Figure 5 – Source Data 9; related to Figure 5–figure supplement 2

Figure 6 – Source Data 1; related to Figure 6D

Figure 7 – Source Data 1; related to Figure 7B

Figure 7 – Source Data 2; related to Figure 7C

Figure 7 – Source Data 3; related to Figure 7E
